# Correction to 8 papers to correct the volume number

**DOI:** 10.1093/lifemedi/lnag031

**Published:** 2026-09-30

This is a correction to the following 8 papers:

https://doi.org/10.1093/lifemedi/lnaf020

https://doi.org/10.1093/lifemedi/lnaf021

https://doi.org/10.1093/lifemedi/lnaf024

https://doi.org/10.1093/lifemedi/lnaf025

https://doi.org/10.1093/lifemedi/lnaf026

https://doi.org/10.1093/lifemedi/lnaf027

https://doi.org/10.1093/lifemedi/lnaf028

https://doi.org/10.1093/lifemedi/lnaf029

In the originally published versions of these manuscripts, the PDF files erroneously listed “Volume XX” instead of “Volume 4”. These errors have been corrected.

